# Bis-pyrimidine acetamides: design, synthesis and biological evaluation

**DOI:** 10.1186/s13065-017-0312-2

**Published:** 2017-08-08

**Authors:** Sanjiv Kumar, Siong Meng Lim, Kalavathy Ramasamy, Mani Vasudevan, Syed Adnan Ali Shah, Balasubramanian Narasimhan

**Affiliations:** 1 Faculty of Pharmaceutical Sciences, Maharshi Dayanand University, Rohtak, 124001 India; 2 Department of Pharmaceutical Sciences, Maharshi Dayanand University, Rohtak, 124001 India; 3 Faculty of Pharmacy, Universiti Teknologi MARA (UiTM), 42300 Bandar Puncak Alam, Selangor Darul Ehsan Malaysia; 4 Collaborative Drug Discovery Research (CDDR) Group, Pharmaceutical Life Sciences Community of Research, Universiti Teknologi MARA (UiTM), 40450 Shah Alam, Selangor Darul Ehsan Malaysia; 5 Department of Pharmacology and Toxicology, College of Pharmacy, Qassim University, Buraidah, 51452 Kingdom of Saudi Arabia; 6 Atta-ur-Rahman Institute for Natural Products Discovery (AuRIns), Universiti Teknologi MARA, Puncak Alam Campus, 42300 Bandar Puncak Alam, Selangor D. E Malaysia

**Keywords:** Bis-pyrimidines, Claisen–Schmidt condensation, Antimicrobial, Anticancer, SAR

## Abstract

**Background:**

In the past few years, increased resistance of microorganisms towards antimicrobial agents become a serious health problem, so there is a need for the discovery of new antimicrobial agents. On the other hand, bis-pyrimidines possess various types of biological activity. In view of this, in the present study we have designed and synthesized a new series of bis-pyrimidine acetamides by Claisen–Schmidt condensation and screened for its in vitro antimicrobial and anticancer activities.

**Results:**

The synthesized bis-pyrimidine acetamide derivatives were confirmed by IR, ^1^H/^13^C-NMR, Mass spectral studies as well C, H, N analyses. The synthesized compounds were evaluated for their in vitro antimicrobial potential against Gram positive (*Staphylococcus aureus* and *Bacillus subtilis*); Gram negative (*Escherichia coli, Pseudomonas aeruginosa* and *Salmonella enterica*) bacterial and fungal (*Candida albicans* and *Aspergillus niger*) strains by tube dilution technique and the minimum inhibitory concentration (MIC) recorded in µmol/mL was comparable to reference drugs, cefadroxil (antibacterial) and fluconazole (antifungal). The in vitro anticancer activity (IC_50_ value) determined against human colorectal carcinoma (HCT116) cancer cell line by Sulforhodamine B (SRB) technique and 5-fluorouracil used as reference drug.

**Conclusions:**

The biological study demonstrated that compounds **3**, **13**, **16**, **17** and **18** were found to be most active antimicrobial agents with best MIC values than the cefadroxil (antibacterial) and fluconazole (antifungal) and compounds **12**, **16** and **18** found to have better anticancer activity against human colorectal carcinoma (HCT116) cancer cell line with best IC_50_ value than the 5-fluorouracil (anticancer).

**Graphical abstract:**
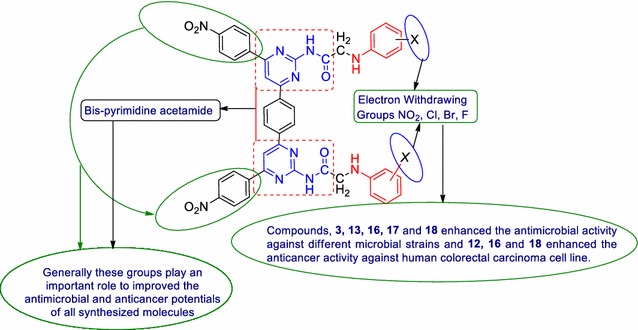
SAR of bis-pyrimidine acetamides

## Background

**Fig. 1:**
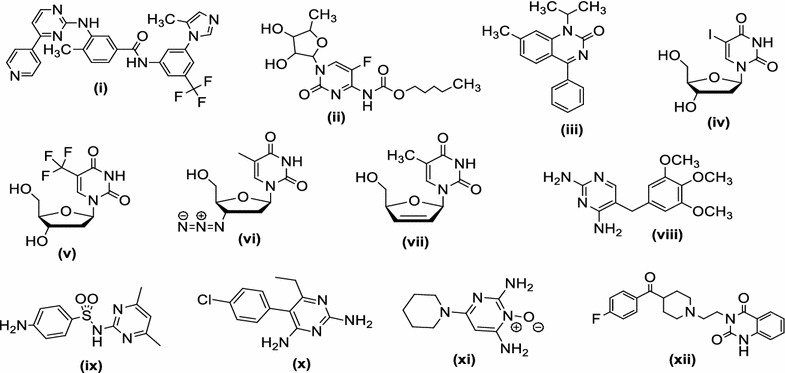
Some marketed drugs contains pyrimidine moiety

**Fig. 2:**
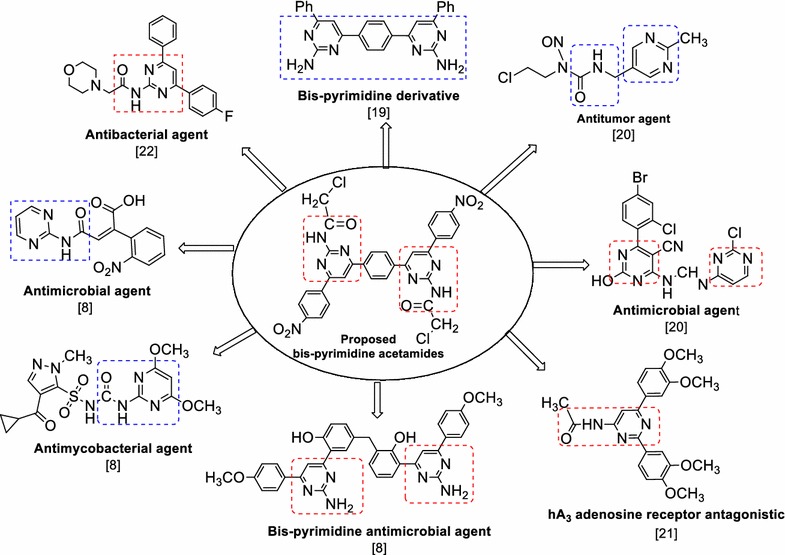
Biological profile of pyrimidine acetamide derivatives found in the resent literature

The treatment of bacterial infections remains a challenging therapeutic problem because of emerging infectious diseases as well the increasing number of multidrug resistant microbial pathogens. Despite the many antimicrobial and chemotherapeutic agents are available in market, the emergence of old and new antibiotic resistant bacterial species in the last decade lead to a substantial need for the discovery of new classes of antimicrobial compounds [1]. Cancer is one of the most serious health problems all over the world and one of the leading causes of death. Thus, in the past for several decades, researchers have been struggling to find effective clinical approaches for the treatment of cancer and search for novel anticancer agents [2]. Among a wide variety of heterocyclic compounds that have been explored for developing medicinally important molecules, nitrogen containing heterocyclic pyrimidine derivatives occupies an important place in the field of medicinal chemistry [3]. The presence of a pyrimidine base in thymine, cytosine and uracil, which are the essential binding blocks of nucleic acids, DNA and RNA is one possible reason for their biological and therapeutical activities. Number of marketed drugs (Fig. 1) contains pyrimidine nucleus i.e. nilotinib (i) and capecitabine (ii) as anticancer; proquazone (iii) as anti-inflammatory; idoxuridine (iv) and trifluoridine (v) as antiviral; zidovudine (vi) and stavudine (vii) as anti-HIV; trimethoprim (viii) and sulphamethiazine (ix) as antibacterial; pyrimethamine (x) as antimalarial; minoxidil (xi) and ketanserin (xii) as antihypertensive [4]. The literature survey indicated that a wide range of pharmacological activities are exhibited by the compounds encompassing pyrimidine nucleus i.e. antibacterial [5], antifungal [6], antileishmanial [7], antimycobacterial [8], antimicrobial [9], anti-inflammatory [10], anticancer [11], antiviral [12], antitubercular [13], antimalarial [14], antioxidant activity [15], central nervous system (CNS) depressant and calcium channel blocker [8]. Based on the literature survey and biological profile of bis-pyrimidine acetamides summarized in Fig. 2 and in continuation of our efforts in searching novel antimicrobial and anticancer agents [16–18], in the present work, we hereby report the synthesis, antimicrobial and anticancer activities of a series of bis-pyrimidine acetamide derivatives.

## Results and discussion

### Chemistry

**Scheme 1:**
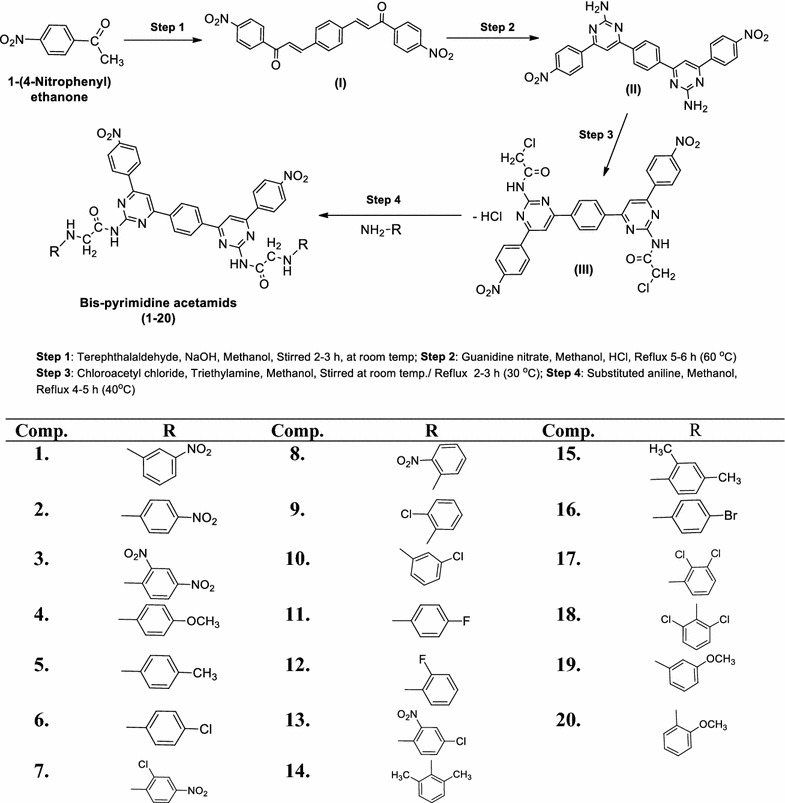
Synthesis of *N, N*′-(6,6′-(1,4-phenylene)bis(4-(4-nitrophenyl)pyrimidine-6,2-diyl)) bis(2-chloroacetamide) analogues

A general approach to synthesize the designed bis-pyrimidine acetamide derivatives based on Claisen–Schmidt condensation is outlined in Scheme 1. Initially, the bis-chalcone (**I**) was synthesized by the reaction of 1-(4-nitrophenyl)ethanone and terephthalaldehyde. The cyclization of a bis-chalcone into the corresponding bis-pyrimidine (**II**) was accomplished by the reaction of the bis-chalcone with guanidine nitrate. The reaction of bis-pyrimidine (**II**) with 2-chloroacetyl chloride resulted in the formation of **III** {*N,N*′-(6,6′-(1,4-phenylene)bis(4-(4-nitrophenyl)pyrimidine-6,2-diyl))bis(2-chloroacetamide)} which on reaction with corresponding substituted aniline yielded the title compounds (**1**–**20**). The chemical structures of the synthesized compounds were established by the determination of their physicochemical and spectroscopic data (FT-IR, ^1^H-NMR, ^13^C-NMR, Mass) and elemental analyses. The structure of the bis-chalcone was confirmed by the corresponding IR (KBr pellets, cm^−1^). The IR spectrum of bis-chalcone (**I)** showed the characteristic band at 1690 cm^−1^ which indicated the presence of a –C=O group and characteristic bands at 3106 and 1517 cm^−1^ indicated the presence of C–H and C=C group in aromatic ring respectively. The existence of Ar–NO_2_ group in bis-chalcone (**I)** was displayed by the existence of symmetric Ar–NO_2_ stretches in the scale of 1341 cm^−1^ and characteristic bands at 2906 and 1598 cm^−1^ indicated the presence of C–H and C=C group in alkyl chain respectively. Bis-pyrimidine (**II)** showed the characteristic bands at 3106 and 1518 cm^−1^ for the presence of C–H and C=C group in aromatic ring respectively and characteristic bands at 3370, 1600 and 1349 cm^−1^ for indicated the presence of C–NH_2_; N=CH str., of pyrimidine and C–N sym stretches of Ar–NO_2_ group. *N,N*′-(6,6′-(1,4-Phenylene)bis(4-(4-nitrophenyl)pyrimidine-6,2-diyl))bis(2-chloroacetamide) **III** showed appearance of IR stretching around 3105 and 1519 cm^−1^ in the spectral data for the presence of C–H and C=C group in aromatic ring respectively and characteristic bands at 1688, 1599, 2928, 761 and 1349 cm^−1^ for the NH–C=O; N=CH str., of pyrimidine; C–H str., CH_2_; C–Cl str., (Ar–Cl); C–N sym str. of aromatic NO_2_ group respectively. The structure of the bis-chalcone and its cyclized products were further confirmed by the corresponding ^1^H-NMR spectra. The ^1^H-NMR spectrum of bis-chalcone **I** showed two doublets at 7.59 ppm (*J* = 15.1 Hz) and 8.06 ppm (*J* = 15.1 Hz); indicating that the CH=CH group in the enone linkage is in a *trans*-conformation. The ^1^H-NMR spectrum of intermediate-**II** showed a multiplet signals between 7.43 and 8.40 δ, ppm confirming the cyclisation of the bis-chalcone to give bis-pyrimidine ring. The ^1^H-NMR spectrum of compound intermediate-**II** showed a sharp singlet at 6.99 δ, ppm due to the NH_2_ protons and it also showed a sharp singlet at 7.86 δ, ppm due to HC=C group, which confirmed the cyclization of the bis-chalcone into a bis-pyrimidine ring. Intermediate (**III**) showed the multiplet signals between 7.54 and 8.93 δ, ppm in ^1^H-NMR spectra which is indicative of aromatic proton and also exhibited sharp singlet at 7.42, 8.10, 3.66 δ, ppm due to the presence of HC=C; –NH; –CH_2_ respectively confirmed the conversion of the bis-pyrimidine into bis-pyrimidine acetamide. The appearance of IR stretching around 1575–1569, 3086–2851 and 1580–1600 cm^−1^ in the spectral data of synthesized derivatives (**1**–**20**) specified the existence of C=N group of pyrimidine ring; C–H and C=C group respectively. The IR absorption band in the scale of 1106–1105 cm^−1^ corresponds to the C–F stretching of aromatic-fluoro compounds (**11** and **12**) and 697–536 cm^−1^ corresponds to the C–Br stretching of aromatic-bromo derivative (**16**). The existence of an arylalkyl ether category (C–O–C, Ar–OCH_3_) in derivatives, **4**, **19** and **20** are established by the existence of an IR symmetric stretches around 1107–1088 cm^−1^. Further, the existence of halogen group in compounds (**6**, **7**, **9**, **10**, **13**, **17** and **18**) is indicated by the existence of Ar–Cl stretching vibrations at 761–700 cm^−1^. The impression of IR stretching at 1692–1620 cm^−1^ in the spectra of specified the existence of NH–C=O group of synthesized derivatives. The multiplet signals between 6.74 and 9.08 δ, ppm in ^1^H-NMR spectra is indicative of aromatic proton of synthesized derivatives (**1**–**20**). The compounds, **4**, **19** and **20** showed singlet at 3.38–3.75 δ, ppm due to the presence of OCH_3_ of Ar–OCH_3_ in their structure. All compounds showed singlet at 7.47–7.91 δ, ppm due to the existence of N=CH in pyrimidine ring. Synthesized compounds showed appearance of IR stretching around 1349 cm^−1^ which indicated the presence of C–N sym str. of aromatic NO_2_ group. Compounds, **5**, **14** and **15** showed singlet at 2.09–2.51 δ, ppm due to existence of –CH_3_ at *ortho* and *para* position. All compounds showed singlet at 3.34–3.38 δ, ppm and 8.00–8.08 δ, ppm due to the existence of –CH_2_– and –NH– groups respectively. The elemental analysis results of the synthesized bis-pyrimidine acetamide derivatives were found within ±0.4% of the theoretical results. Finally, the ^13^C-NMR spectra of the bis-chalcone and the cyclized bis-pyrimidine were recorded in DMSO-*d*_6_ and the spectral signals were in good agreement with the proposed molecular structure of the synthesized compounds. ^13^C-NMR spectra details of all compounds are given in the experimental part. Characteristic molecular ion peaks were observed in the mass spectra of the bis-chalcone and the cyclized bis-pyrimidine and final bis-pyrimidine acetamide derivatives.

#### Antimicrobial activity

**Fig. 3:**
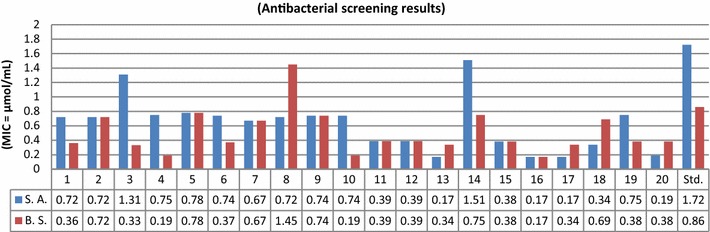
Antibacterial screening results against Gram positive species

**Fig. 4:**
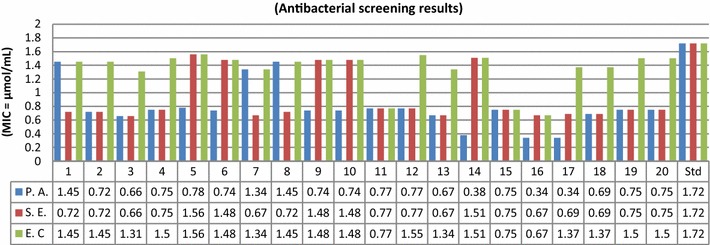
Antibacterial screening results against Gram negative species

**Fig. 5:**
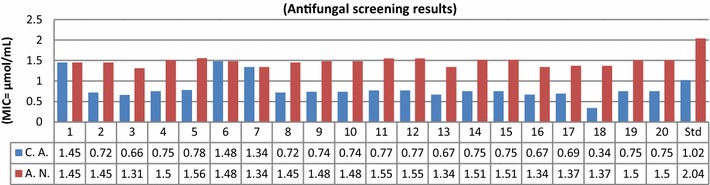
Antifungal screening results against fungal species

The synthesized compounds were screened for their in vitro antimicrobial activity by tube dilution method. The investigation of antimicrobial screening revealed that some of the synthesized compounds showed moderate to good antimicrobial activity against Gram positive bacterial species: *Staphylococcus aureus*, *Bacillus subtilis* and Gram negative bacterial species: *Escherichia coli, Pseudomonas aeruginosa*, *Salmonella enterica* and fungal species: *Aspergillus niger* and *Candida albicans.* Particularly, compounds **3**, **13**, **16**, **17** and **18** have shown more promising antimicrobial activity as compared to standard drugs cefadroxil (antibacterial) and fluconazole (antifungal). The remaining compounds displayed average to poor activity against all seven microbial species. In vitro antimicrobial activity results of synthesized compound are summarized in Table 1. Antibacterial screening results (Fig. 3) against Gram positive bacterial species demonstrated that compound **16** has showed good antibacterial activity against *S. aureus* and *B. subtilis* respectively. Compounds, **13** and **17** have sensible activity against *S. aureus.* The MIC values of **13** nearly close to MIC value of standard drugs cefadroxil while **16** and **17** have more active than standard drugs. Antibacterial screening results (Fig. 4) indicated that compound **3** possessed excellent activity against *S. enterica*. Compound **16** exhibited promising activity against *P. aeruginosa* and *E. coli*. Compound **17** was most active against *P. aeruginosa.* The MIC values of compounds **16** and **17** was more than the MIC values of standard drug cefadroxil Gram negative bacterial species. Antifungal screening results (Fig. 5) indicated that compound **3** and **18 **were found to be most effective ones against *A. niger* and *C. albicans* respectively. The rest of the compounds of the series exhibited average to poor antifungal activity. The antimicrobial activity results of synthesized bis-pyrimidine acetamide derivatives indicated that the synthesized compounds are more active than the standard drugs and may be taken as a lead compound to discover novel antimicrobial agents.

**Table 1:** Antimicrobial and anticancer screening results of synthesized compounds

Compounds No.	Minimum inhibitory concentration (MIC = µmol/mL)							IC_50_ (µmol/mL) Cancer cell line
	Bacterial species					Fungal species		
	Gram positive		Gram negative					
	*S. aureus* MTCC-3160	*B. subtilis* MTCC-441	*P. aeruginosa* MTCC-3542	*S. enterica* MTCC-1165	*E. coli* MTCC-443	*C. albicans* MTCC-227	*A. niger* MTCC-281	HCT-116
1.	0.72	0.36	1.45	0.72	1.45	1.45	1.45	3.86
2.	0.72	0.72	0.72	0.72	1.45	0.72	1.45	3.86
3.	1.31	0.33	0.66	0.66	1.31	0.66	1.31	3.50
4.	0.75	0.19	0.75	0.75	1.50	0.75	1.50	4.00
5.	0.78	0.78	0.78	1.56	1.56	0.78	1.56	4.16
6.	0.74	0.37	0.74	1.48	1.48	1.48	1.48	3.96
7.	0.67	0.67	1.34	0.67	1.34	1.34	1.34	3.58
8.	0.72	1.45	1.45	0.72	1.45	0.72	1.45	3.86
9.	0.74	0.74	0.74	1.48	1.48	0.74	1.48	1.98
10.	0.74	0.19	0.74	1.48	1.48	0.74	1.48	3.96
11.	0.39	0.39	0.77	0.77	0.77	0.77	1.55	1.52
12.	0.39	0.39	0.77	0.77	1.55	0.77	1.55	0.74
13.	0.17	0.34	0.67	0.67	1.34	0.67	1.34	3.58
14.	1.51	0.75	0.38	1.51	1.51	0.75	1.51	4.02
15.	0.38	0.38	0.75	0.75	0.75	0.75	1.51	2.17
16.	0.17	0.17	0.34	0.67	0.67	0.67	1.34	0.98
17.	0.17	0.34	0.34	0.69	1.37	0.69	1.37	1.22
18.	0.34	0.69	0.69	0.69	1.37	0.34	1.37	0.73
19.	0.75	0.38	0.75	0.75	1.50	0.75	1.50	4.00
20.	0.19	0.38	0.75	0.75	1.50	0.75	1.50	2.16
DMSO	0.00	0.00	0.00	0.00	0.00	0.00	0.00	–
Cefadroxil	1.72	0.86	1.72	1.72	1.72	–	–	–
Fluconazole	–	–	–	–	–	1.02	2.04	–
5-Fluorouracil	–	–	–	–	–	–	–	7.67

Std. drugs: cefadroxil—Antibacterial; fluconazole—Antifungal; 5-fluorouracil—Anticancer

#### Anticancer activity

**Fig. 6:**
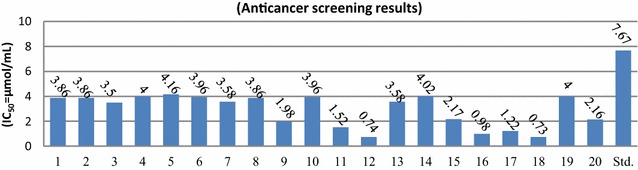
Anticancer screening results of the synthesized compounds

The anticancer screening (IC_50_ = µmol/mL) of the synthesized bis-pyrimidine acetamide derivatives was determined against human colorectal carcinoma [HCT-116 (ATCC CCL-247)] cancer cell line by Sulforhodamine B (SRB) technique [28] using 5-fluorouracil as reference drug and the results are presented in Table 1. Anticancer screening results (Fig. 6) revealed that in general bis-pyrimidine acetamides exhibited good anticancer potential against human colorectal cancer cell line, especially, Compounds, **12**, **16** and **18** displayed more anticancer activity than the reference drug 5-fluorouracil (IC_50_ = 7.67 µmol/mL) with IC_50_ values of 0.74, 0.98 and 0.73 µmol/mL respectively.

**Fig. 7:**
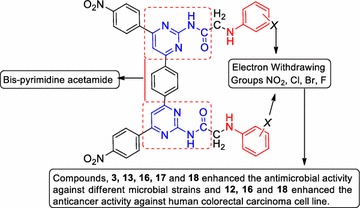
Structure activity relationship of bis-pyrimidine acetamides

The structure–activity relationship of the synthesized bis-pyrimidine acetamides indicated that the compounds bearing electron withdrawing group at different position of phenyl group plays an important role in enhancing the antimicrobial and anticancer potentials (Fig. 7).

### Experimental part

Preparatory materials for the research work were obtained from commercial sources and were used without further purification. All reactions were monitored by thin-layer chromatography on 0.25 mm silica gel (Merck) plates, using benzene as mobile phase and spots were observed by exposure to iodine vapours or visualized with UV light. Melting points of synthesized compounds was determined in open capillary tube. An infrared spectrum was recorded (KBr-pellets) in Bruker 12060280, Software: OPUS 7.2.139.1294 spectrometer. ^1^H-NMR and ^13^C-NMR were recorded at 600 and 150 MHz, respectively on Bruker Avance III 600 NMR spectrometer by appropriate deuterated solvents. The results are conveyed in parts per million (*δ*, ppm) downfield from tetramethyl silane (internal standard). ^1^H-NMR spectral details of the synthesized derivatives are represented with multiplicity like singlet (s); doublet (d); triplet (t); multiplet (m) and the number hydrogen ion. Elemental analysis of the new synthesized compounds was obtained by Perkin–Elmer 2400 C, H and N analyzer. All the compounds gave C, H and N analysis within ±0.4% of the theoretical results. Mass spectra were taken on Waters Micromass Q-ToF Micro instrument. The synthesized compounds were characterized by the determination of their physicochemical and spectral characteristics.

#### General procedure of the synthesized bis-pyrimidine acetamide derivatives (1–20)

##### Step i: Synthesis of 3,3′-(1,4-phenylene)bis(1-(4-nitrophenyl)prop-2-en-1-one) (I)

A mixture of 1-(4-nitrophenyl)ethanone (0.02 mol) and terephthalaldehyde (0.01 mol) in methanol (5–10 mL) was stirred with drop wise addition of 40% sodium hydroxide solution (10 mL) at room temperature till a dark yellow mass was obtained (2–3 h). Then reaction mixture was allowed to stand overnight at room temperature and then was poured into icecold water and acidified with hydrochloric acid and the precipitated 3,3′-(1,4-phenylene)bis(1-(4-nitrophenyl) prop-2-en-1-one) was filtered, dried and recrystallized from methanol [23, 24].

##### Step ii: Synthesis of 6,6′-(1,4-phenylene)bis(4-(4-nitrophenyl)pyrimidin-2-amine) (II)

The solution of 3,3′-(1,4-phenylene)bis(1-(4-nitrophenyl)prop-2-en-1-one) (0.01 mol) (*synthesized in previous step*-*i*) in methanol (80 mL) was added with 0.01 mol of potassium hydroxide and 40 mL of 0.50 M solution of guanidine nitrate and refluxed for 5–6 h. The reaction mixture was then cooled and acidified with few drops of hydrochloric acid (20 mL of 0.5 M solution) and the resultant precipitate, 6,6′-(1,4-phenylene)bis(4-(4-nitrophenyl) pyrimidin-2-amine) was separated dried and recrystallized from methanol [23, 24].

##### Step iii: Synthesis of N,N′-(6,6′-(1,4-phenylene)bis(4-(4-nitrophenyl)pyrimidine-6,2-diyl))bis (2-chloroacetamide) (III)

In ethanol (30 mL), chloroacetyl chloride (0.02 mol) and 2–3 drops of triethylamine were added and the mixture was stirred in water bath for 10 min after that the solution of 6,6′-(1,4-phenylene)bis(4-(4-nitrophenyl)pyrimidin-2-amine) (*synthesized in previous step*-*ii*) (0.01 mol) in ethanol (80 mL) was added drop wise and refluxed for 2–3 h. The reaction mixture was then cooled and poured into icecold water and resultant precipitate was filtered and washed with water, dried and recrystallized from alcohol [25].

##### Step iv: Synthesis of final (1–20) bis-pyrimidine acetamide derivatives

The reaction mixture of *N,N*′-(6,6′-(1,4-phenylene)bis(4-(4-nitrophenyl)pyrimidine-6,2-diyl))bis(2-chloroacetamide) (0.01 mol) and substituted aniline (0.02 mol) in ethanol was refluxed for 4–5 h. The reaction progress was monitored by thin layer chromatography. After completion of reaction, the reaction mixture was poured into icecold water and the precipitated title compound was filtered, dried and recrystallized from methanol.

#### Spectral data interpretation of the synthesized compounds (Fig. 8)

##### 3,3′-(1,4-Phenylene)bis(1-(4-nitrophenyl)prop-2-en-1-one) (I)

IR (KBr pellets, cm^−1^): 3106 (C–H str., phenyl nucleus), 1517 (C=C str., phenyl nucleus), 1690 (C=O str.), 1598 (C=C str., alkyl chain), 2906 (C–H str., alkyl chain), 1341 (C–N sym str., Ar–NO_2_); ^1^H-NMR (δ, DMSO-*d*_6_): 6.94–9.08 {m, 12H, Ar = H-2, H-3, H-5, H-6, (H-2″, H-3″, H-5″, H-6″) × 2}, 7.59 {d, 4H, (CH)_2_, *J* = 15.1 Hz), 8.06 (d, 4H, (CH)_2_, *J* = 15.1 Hz of CH_2_ = CH_2_}; ^13^C-NMR (δ, DMSO-*d*_6_): 129.8 (C-2, C-3, C-5, C-6), 133.4 (C-1, C-4); 148.0 (C-1′), 121.3 (C-2′), 164.7 (C = O); 145.1 (C-1″), 130.0 (C-2″, C-6″), 124.9 (C-3″, C-5″), 162.5 (C-4″); Elem. Anal. Calcd. C_24_H_16_N_2_O_6_: C, 67.29; H, 3.76; N, 6.54; Found: C, 67.26; H, 3.75; N, 6.50; MS ES+(ToF): *m/z* 429 [M^+^ +1].

**Fig. 8:**
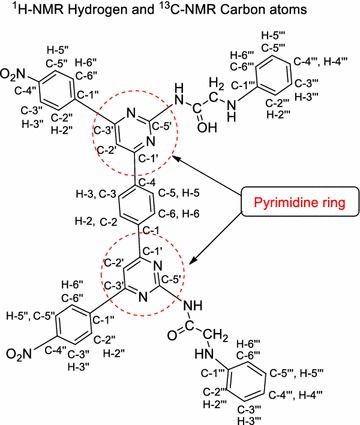
Number of hydrogen and carbon atoms is present in the synthesized compounds

##### 6,6′-(1,4-Phenylene)bis(4-(4-nitrophenyl)pyrimidin-2-amine) (II)

IR (KBr pellets, cm^−1^): 3107 (C–H str., phenyl nucleus), 1518 (C=C str., phenyl nucleus), 3370 (C–NH_2_ str.), 1600 (N=CH str., of pyrimidine), 1349 (C–N sym str., Ar–NO_2_); ^1^H-NMR (δ, DMSO-*d*_6_): 7.43–8.40 {m, 12H, Ar = H-2, H-3, H-5, H-6, (H-2″, H-3″, H-5″, H-6″) × 2}, 7.86 (s, 2H, (CH)_2_ of pyrimidine), 6.97 (s, 2H, (NH)_2_); ^13^C-NMR (δ, DMSO-*d*_6_): 128.5 (C-2, C-3, C-5, C-6), 134.6 (C-1, C-4); 157.8 (C-1′, C-3′, C-5′), 102 (C-2′), 149.7 (C-5′) pyrimidine; 141.5 (C-1″), 126.4 (C-2″, C-6″), 124.4 (C-3″, C-5″), 148.3 (C-4″); Elem. Anal. Calcd. C_26_H_18_N_8_O_4_: C, 61.66; H, 3.58; N, 22.12; Found: C, 61.64; H, 3.51; N, 22.15; MS ES + (ToF): *m/z* 507 [M^+^+1].

##### N,N′-(6,6′-(1,4-Phenylene)bis(4-(4-nitrophenyl)pyrimidine-6,2-diyl))bis(2-chloroacetamide) (III)

IR (KBr pellets, cm^−1^): 3105 (C–H str., phenyl nucleus), 1519 (C=C str., phenyl nucleus), 1688 (NH–C=O str.), 1599 (N=CH str., of pyrimidine), 2928 (C–H str., CH_2_), 761 (C–Cl str., Ar–Cl), 1349 (C–N sym. str., Ar–NO_2_); ^1^H-NMR (δ, DMSO-*d*_6_): 7.54–8.93 {m, 12H, Ar = H-2, H-3, H-5, H-6, (H-2″, H-3″, H-5″, H-6″) × 2}, 7.42 (s, 2H, (CH)_2_ of pyrimidine), 8.10 (s, 2H, (NH)_2_), 3.66 (s, 4H, (CH_2_)_2_); ^13^C-NMR (δ, DMSO-*d*_6_): 128.5 (C-2, C-3, C-5, C-6), 136.6 (C-1, C-4); 162.0 (C-1′, C-3′), 108.0 (C-2′), 149.7 (C-5′) pyrimidine; 141.0 (C-1″), 126.6 (C-2″, C-6″), 124.5 (C-3″, C-5″), 149.8 (C-4″), 188.0 (C = O), 44.3(CH_2_); Elem. Anal. Calcd. C_30_H_20_Cl_2_N_8_O_6_: C, 54.64; H, 3.06; N, 16.99; Found: C, 54.60; H, 3.00; N, 16.92; MS ES + (ToF): *m/z* 660 [M^+^ +1].

##### N,N′-(6,6′-(1,4-Phenylene)bis(4-(4-nitrophenyl)pyrimidine-6,2-diyl))bis(2-((3-nitrophenyl) amino)acetamide) (1)

Brown crystals; Yield: 70.12%; M.p.: 252–254 °C; R*f* value: 90.12; IR (KBr, cm^−1^): 3085 (C–H str., phenyl nucleus), 1528 (C=C str., phenyl nucleus), 1679 (NH–C=O str.), 1572 (N=CH str., of pyrimidine), 2863 (C–H str., CH_2_), 1217 (C–NH–str.), 1347 (C–N sym. str., Ar–NO_2_); ^1^H-NMR (δ, DMSO-*d*_6_): 7.63–8.25 {m, 20H, Ar = H-2, H-3, H-5, H-6, (H-2″, H-3″, H-5″, H-6″) × 2, (H-2‴, H-4‴,H-5‴, H-6‴) × 2}, 7.63 (s, 2H, (CH)_2_ of pyrimidine), 8.08 (s, 2H, (NH)_2_), 4.0 (s, 2H, (NH)_2_), 3.40 (s, 4H, (CH_2_)_2_); ^13^C-NMR (δ, DMSO-*d*_6_): 128.3, 128.4 (C-2, C-3, C-5, C-6), 136.5 (C-1, C-4); 165.0 (C-1′, C-3′), 109 (C-2′), 149.7 (C-5′) pyrimidine; 141.0 (C-1″), 126.5 (C-2″, C-6″), 124.5 (C-3″, C-5″), 149.6 (C-4″); 149.5 (C-1‴, C-3‴), 106.9 (C-2‴), 129.9 (C-5‴), 122.1 (C-6‴); 170.2 (C = O), 48.0 (CH_2_); Elem. Anal. Calcd. C_42_H_30_N_12_O_10_: C, 58.47; H, 3.50; N, 19.48; Found: C, 58.42; H, 3.49; N, 19.46; MS ES + (ToF): *m/z* 864 [M^+^ +1].

##### N,N′-(6,6′-(1,4-Phenylene)bis(4-(4-nitrophenyl)pyrimidine-6,2-diyl))bis(2-((4-nitrophenyl) amino)acetamide) (2)

Dark brown crystals; M.p.: 262–264 °C; R*f* value: 0.32; Yield: 92.45%; IR (KBr, cm^−1^): 3086 (C–H str., phenyl nucleus), 1530 (C=C str., phenyl nucleus), 1679 (NH–C = O str.), 1573 (N=CH str., of pyrimidine), 2929 (C–H str., CH_2_), 1219 (C–NH– str.), 1348 (C–N sym. str., Ar–NO_2_); ^1^H-NMR (δ, DMSO-*d*_6_): 6.74–9.09 (m, 20H, Ar = H-2, H-3, H-5, H-6, (H-2″, H-3″, H-5″, H-6″) × 2, (H-2‴, H-3‴, H-5‴, H-6‴) × 2}, 7.04 (s, 2H, CH of pyrimidine), 8.01 (s, 2H, (NH)_2_), 3.37 (s, 4H, (CH_2_)_2_); ^13^C-NMR (δ, DMSO-*d*_6_): 127.3 (C-2, C-3, C-5, C-6), 138.9 (C-1, C-4); 164.0 (C-1′, C-3′), 112 (C-2′), 148.3 (C-5′) pyrimidine; 126.3 (C-2″, C-6″), 121.5 (C-3″, C-5″), 147.6 (C-4″); 152.5 (C-1‴), 115.3 (C-2‴, C-6‴), 127.5 (C-3‴, C-5‴), 135.6 (C-4‴), 168.2 (C=O), 45.0 (CH_2_); Elem. Anal. Calcd. C_42_H_30_N_12_O_10_: C, 58.47; H, 3.50; N, 19.48; Found: C, 58.41; H, 3.48; N, 19.46; MS ES + (ToF): *m/z* 864 [M^+^ +1].

##### N,N′-(6,6′-(1,4-Phenylene)bis(4-(4-nitrophenyl)pyrimidine-6,2-diyl))bis(2-((2,4-dinitrophenyl) amino)acetamide) (3)

Brown crystals; M.p.: 257–259 °C; R*f* value: 0.41; Yield: 92.67%; IR (KBr pellets, cm^−1^): 3211 (C–H str., phenyl nucleus), 1529 (C=C str., phenyl nucleus), 1620 (NH–C=O str.), 1675 (N=CH str., of pyrimidine), 3086 (C–H str., CH_2_), 1274 (C–NH– str.), 1345 (C–N sym. str., Ar–NO_2_); ^1^H-NMR (δ, DMSO-*d*_6_): 6.95–8.90 {m, 18H, Ar = H-2, H-3, H-5, H-6, (H-2″, H-3″, H-5″, H-6″) × 2, (H-3‴, H-5‴, H-6‴) × 2}, 7.87 (s, 2H, (CH_)2_ of pyrimidine), 8.01 (s, 2H, (NH)_2_), 3.40 (s, 4H, (CH_2_)_2_); ^13^C-NMR (δ, DMSO-*d*_6_): 128.6 (C-2, C-3, C-5, C-6), 135.05 (C-1, C-4); 164.0 (C-1′, C-3′), 102 (C-2′), 149.7 (C-5′) pyrimidine; 141.0 (C-1″), 127.3 (C-2″, C-6″), 124.9 (C-3″, C-5″), 148.6 (C-4″); 152.5 (C-1‴), 135.6 (C-2‴), 119.7 (C-3‴), 138.9 (C-4‴), 130.2 (C-5‴); 168.2 (C=O), 51.0 (CH_2_); Elem. Anal. Calcd. C_42_H_28_N_14_O_14_: C, 52.95; H, 2.96; N, 20.58; Found: C, 52.85; H, 2.46; N, 20.48; MS ES + (ToF): *m/z* 954 [M^+^ +1].

##### N,N′-(6,6′-(1,4-Phenylene)bis(4-(4-nitrophenyl)pyrimidine-6,2-diyl))bis(2-((4-methoxyphenyl) amino)acetamide) (4)

Brown crystals; M.p.: 215–217 °C; R*f* value: 0.53; Yield: 89.44%; IR (KBr pellets, cm^−1^): 3084 (C–H str., phenyl nucleus), 1529 (C=C str., phenyl nucleus), 1680 (NH–C=O str.), 1572 (N=CH str., of pyrimidine), 1221 (C–NH– str.), 1347 (C–N sym. str., Ar–NO_2_), 1088 (C–O–C str., –OCH_3_); ^1^H-NMR (δ, DMSO-*d*_6_): 7.04–9.09 {m, 20H, Ar = H-2, H-3, H-5, H-6, (H-2″, H-3″, H-5″, H-6″) × 2, (H-2‴, H-3‴, H-5‴, H-6‴) × 2}, 7.75 (s, 2H, (CH)_2_ of pyrimidine), 8.0 (s, 2H, (NH)_2_), 3.38 (s, 6H, (OCH_3_)_2_); ^13^C-NMR (δ, DMSO-*d*_6_): 127.3 (C-2, C-3, C-5, C-6), 138.5 (C-1, C-4); 164.0 (C-1′, C-3′), 108 (C-2′), 148.7 (C-5′) pyrimidine; 142.0 (C-1″), 127.5 (C-2″, C-6″), 124.9 (C-3″, C-5″), 149.6 (C-4″); 138.5 (C-1‴), 115.3 (C-2‴, C-6‴), 115.0 (C-3‴, C-5‴), 171.2 (C=O), 51.0 (CH_2_), 53.0 (*p*-OCH_3_); Elem. Anal. Calcd. C_44_H_36_N_10_O_8_: C, 63.46; H, 4.36; N, 16.82; Found: C, 63.44; H, 4.37; N, 16.85; MS ES + (ToF): *m/z* 834 [M^+^ +1].

##### N,N′-(6,6′-(1,4-Phenylene)bis(4-(4-nitrophenyl)pyrimidine-6,2-diyl))bis(2-(p-tolylamino) acetamide) (5)

Brown crystals; M.p.: 250–252 °C; R*f* value: 0.30; Yield: 85.43%; IR (KBr pellets, cm^−1^): 3084 (C–H str., phenyl nucleus), 1529 (C=C str., phenyl nucleus), 1681 (NH–C=O str.), 1569 (N=CH str., of pyrimidine), 2866 (C–H str., CH_2_), 1217 (C–NH– str.), 1348 (C–N sym. str., Ar–NO_2_); ^1^H-NMR (δ, DMSO-*d*_6_): 6.99–8.56 {m, 20H, Ar = H-2, H-3, H-5, H-6, (H-2″, H-3″, H-5″, H-6″) × 2, (H-2‴, H-3‴, H-5‴, H-6‴) × 2}, 7.48 (s, 2H, (CH)_2_ of pyrimidine), 8.03 (s, 2H, (NH)_2_), 2.51 (s, 6H, (CH_3_)_2_); ^13^C-NMR (δ, DMSO-*d*_6_): 128.8, 128.4 (C-2, C-3, C-5, C-6), 136.8, (C-1, C-4); 168.0 (C-1′, C-3′), 108 (C-2′), 149.7 (C-5′) pyrimidine; 141.0 (C-1″), 126.4 (C-2″, C-6″), 123.8 (C-3″, C-5″), 149.7 (C-4″); 144.5 (C-1‴), 117.0 (C-2‴, C-6‴), 129.6 (C-3‴, C-5‴), 188.0 (C=O), 48.0 (CH_2_), 20.5 (*p*-CH_3_); Elem. Anal. Calcd. C_44_H_36_N_10_O_6_: C, 65.99; H, 4.53; N, 17.49; Found: C, 65.92; H, 4.49; N, 17.40; MS ES + (ToF): *m/z* 802 [M^+^ +1].

##### N,N′-(6,6′-(1,4-Phenylene)bis(4-(4-nitrophenyl)pyrimidine-6,2-diyl))bis(2-((4-chlorophenyl) amino)acetamide) (6)

Brown crystals; M.p.: 168–170 °C; R*f* value: 0.42; Yield: 87.58%; IR (KBr pellets, cm^−1^): 3084 (C–H str., phenyl nucleus), 1530 (C=C str., phenyl nucleus), 1687 (NH–C=O str.), 1603 (N=CH str., of pyrimidine), 2866 (C–H str., CH_2_), 1215 (C–NH– str.), 1348 (C–N sym. str., Ar–NO_2_), 739 (C–Cl str., Ar–Cl); ^1^H-NMR (δ, DMSO-*d*_6_): 6.35–9.08 {m, 20H, Ar = H-2, H-3, H-5, H-6, (H-2″, H-3″, H-5″, H-6″) × 2, (H-2‴, H-3‴, H-5‴, H-6‴) × 2}, 7.49 (s, 2H, (CH)_2_ of pyrimidine), 8.00 (s, 2H, (NH)_2_), 3.36 (s, 4H, (CH_2_)_2_); ^13^C-NMR (δ, DMSO-*d*_6_): 128.4 (C-2, C-3, C-5, C-6), 134.5 (C-1, C-4); 164.0 (C-1′, C-3′), 106 (C-2′), 148.3 (C-5′) pyrimidine; 127.4 (C-2″, C-6″), 124.9 (C-3″, C-5″), 147.6 (C-4″); 145.5 (C-1‴), 115.1 (C-2‴, C-6‴), 129.9 (C-3‴, C-5‴), 126.1 (C-4‴); 165.2 (C=O), 53.0 (CH_2_); Elem. Anal. Calcd. C_42_H_30_Cl_2_N_10_O_6_: C, 59.94; H, 3.59; N, 16.64; Found: C, 59.90; H, 3.51; N, 16.60; MS ES + (ToF): *m/z* 843 [M^+^ +1].

##### N,N′-(6,6′-(1,4-Phenylene)bis(4-(4-nitrophenyl)pyrimidine-6,2-diyl))bis(2-((2-chloro-4-nitro phenyl)amino)acetamide) (7)

Brown yellow crystals; M.p.: 170–172 °C; R*f* value: 0.60; Yield: 87.12%; IR (KBr pellets, cm^−1^): 3085 (C–H str., phenyl nucleus), 1530 (C=C str., phenyl nucleus), 1688 (NH–C=O str.), 1603 (N=CH str., of pyrimidine), 2869 (C–H str., CH_2_), 1215 (C–NH– str.), 1348 (C–N sym. str., Ar–NO_2_), 740 (C–Cl str., Ar–Cl); ^1^H-NMR (δ, DMSO-*d*_6_): 6.48–9.08 {m, 18H, Ar = H-2, H-3, H-5, H-6, (H-2″, H-3″, H-5″, H-6″) × 2, (H-3‴, H-5‴, H-6‴) × 2}, 7.87 (s, 2H, (CH)_2_ of pyrimidine), 8.01 (s, 2H, (NH)_2_), 3.37 (s, 4H, (CH_2_)_2_); ^13^C-NMR (δ, DMSO-*d*_6_): 129.3 (C-2, C-3, C-5, C-6), 135.8 (C-1, C-4); 163.0 (C-1′, C-3′), 113 (C-2′), 148.7 (C-5′) pyrimidine; 126.3 (C-2″, C-6″), 124.9 (C-3″, C-5″), 149.3 (C-4″); 151.3 (C-1‴), 124.3 (C-2‴), 138.9 (C-4‴), 125.1 (C-3‴), 122.1 (C-5‴); 166.2 (C=O), 51.0 (CH_2_); Elem. Anal. Calcd. C_42_H_28_Cl_2_N_12_O_10_: C, 54.15; H, 3.03; N, 18.04; Found: C, 54.11; H, 3.00; N, 18.12; MS ES + (ToF): *m/z* 933 [M^+^ +1].

##### N,N′-(6,6′-(1,4-Phenylene)bis(4-(4-nitrophenyl)pyrimidine-6,2-diyl))bis(2-((2-nitrophenyl) amino)acetamide) (8)

Light Brown crystals; M.p.: 163–165 °C; R*f* value: 0.34; Yield: 84.23%; IR (KBr pellets, cm^−1^): 3085 (C–H str., phenyl nucleus), 1530 (C=C str., phenyl nucleus), 1688 (NH–C=O str.), 1603 (N=CH str., of pyrimidine), 2868 (C–H str., CH_2_), 1215 (C–NH– str.), 1348 (C–N sym. str., Ar–NO_2_); ^1^H-NMR (δ, DMSO-*d*_6_): 7.03-8.25 {m, 20H, Ar = H-2, H-3, H-5, H-6, (H-2″, H-3″, H-5″, H-6″) × 2, (H-3‴, H-4‴, H-5‴, H-6‴) × 2}, 7.79 (s, 2H, (CH)_2_ of pyrimidine), 8.04 (s, 2H, (NH)_2_), 3.38 (s, 4H, (CH_2_)_2_); ^13^C-NMR (δ, DMSO-*d*_6_): 127.4 (C-2, C-3, C-5, C-6), 135.6 (C-1, C-4); 162.0 (C-1′, C-3′), 105 (C-2′), 148.7 (C-5′) pyrimidine; 141.0 (C-1″), 126.5 (C-2″, C-6″), 124.3 (C-3″, C-5″), 149.6 (C-4″); 147.5 (C-1‴), 130.1 (C-2‴), 125.9 (C-3‴), 119.1 (C-4‴), 136.2 (C-5‴); 164.2 (C=O), 50.0 (CH_2_); Elem. Anal. Calcd. C_42_H_30_N_12_O_10_: C, 58.47; H, 3.50; N, 19.48; Found: C, 58.42; H, 3.45; N, 19.41; MS ES + (ToF): *m/z* 864 [M^+^ +1].

##### N,N′-(6,6′-(1,4-Phenylene)bis(4-(4-nitrophenyl)pyrimidine-6,2-diyl))bis(2-(2-chlorophenyl) acetamide) (9)

Canary yellow crystals; M.p.: 150–152 °C; R*f* value: 0.58; Yield: 75.25%; IR (KBr pellets, cm^−1^): 3075 (C–H str., phenyl nucleus), 1521 (C=C str., phenyl nucleus), 1690 (NH–C=O str.), 1598 (N=CH str., of pyrimidine), 2851 (C–H str., CH_2_), 1210 (C–NH– str.), 1344 (C–N sym. str., Ar–NO_2_), 757 (C–Cl str., Ar–Cl); ^1^H-NMR (δ, DMSO-*d*_6_): 7.80–8.25 {m, 20H, Ar = H-2, H-3, H-5, H-6, (H-2″, H-3″, H-5″, H-6″) × 2, (H-3‴, H-4‴, H-5‴, H-6‴) × 2}, 7.80 (s, 2H, (CH)_2_ of pyrimidine), 8.00 (s, 2H, (NH)_2_), 3.37 (s, 4H, (CH_2_)_2_); ^13^C-NMR (δ, DMSO-*d*_6_): 128.6 (C-2, C-3, C-5, C-6), 135.5 (C-1, C-4); 163.0 (C-1′, C-3′), 107 (C-2′), 149.7 (C-5′) pyrimidine; 141.0 (C-1″), 127.5 (C-2″, C-6″), 123.5 (C-3″, C-5″), 149.8 (C-4″); 134.5 (C-1‴), 129.1 (C-2‴, C-3‴, C-4‴), 127.9 (C-5‴), 133.1 (C-6‴); 156.2 (C=O), 39 (CH_2_); Elem. Anal. Calcd. C_42_H_30_Cl_2_N_10_O_6_: C, 59.94; H, 3.59; N, 16.64; Found: C, 59.90; H, 3.53; N, 16.61; MS ES + (ToF): *m/z* 843 [M^+^ +1].

##### N,N′-(6,6′-(1,4-Phenylene)bis(4-(4-nitrophenyl)pyrimidine-6,2-diyl))bis(2-(2-chlorophenyl) acetamide) (10)

Light yellow crystals; M.p.: 138–140 °C; R*f* value: 0.31; Yield: 80.22%; IR (KBr pellets, cm^−1^): 3074 (C–H str., phenyl nucleus), 1521 (C=C str., phenyl nucleus), 1690 (NH–C=O str.), 1597 (N=CH str., of pyrimidine), 2854 (C–H str., CH_2_), 1210 (C–NH– str.), 1344 (C–N sym. str., Ar–NO_2_), 757 (C–Cl str., Ar–Cl); ^1^H-NMR (δ, DMSO-*d*_6_): 7.82 {m, 20H, Ar = H-2, H-3, H-5, H-6, (H-2″, H-3″, H-5″, H-6″) × 2, (H-2‴, H-4‴, H-5‴, H-6‴) × 2}, 7.80 (s, 2H, (CH)_2_ of pyrimidine), 8.00 (s, 2H, (NH)_2_), 3.38 (s, 4H, (CH_2_)_2_); ^13^C-NMR (δ, DMSO-*d*_6_): 128.3 (C-2, C-3, C-5, C-6), 135.5 (C-1, C-4); 165.0 (C-1′, C-3′), 108 (C-2′), 149.8 (C-5′) pyrimidine; 142.0 (C-1″), 127.5 (C-2″, C-6″), 123.5 (C-3″, C-5″), 149.6 (C-4″); 149.5 (C-1‴), 110 (C-2‴), 134.9 (C-3‴), 120.4 (C-5‴); 163.2 (C=O), 56.0 (CH_2_); Elem. Anal. Calcd. C_42_H_30_Cl_2_N_10_O_6_: C, 59.94; H, 3.59; N, 16.64; Found: C, 59.90; H, 3.52; N, 16.67; MS ES + (ToF): *m/z* 843 [M^+^ +1].

##### N,N′-(6,6′-(1,4-Phenylene)bis(4-(4-nitrophenyl)pyrimidine-6,2-diyl))bis(2-((4-fluorophenyl) amino)acetamide) (11)

Yellow crystals; M.p.: 123–125 °C; R*f* value: 0.33; Yield: 85.63%; IR (KBr pellets, cm^−1^): 3073 (C–H str., phenyl nucleus), 1518 (C=C str., phenyl nucleus), 1665 (NH–C=O str.), 1596 (N=CH str., of pyrimidine), 2852 (C–H str., CH_2_), 1210 (C–NH– str.), 1338 (C–N sym. str., Ar–NO_2_), 1105 (C–F str., Ar–F); ^1^H-NMR (δ, DMSO-*d*_6_): 7.25–8.69 {m, 20H, Ar = H-2, H-3, H-5, H-6, (H-2″, H-3″, H-5″, H-6″) × 2, (H-2‴, H-3‴, H-5‴, H-6‴) × 2}, 7.39 (s, 2H, (CH)_2_ of pyrimidine), 8.01 (s, 2H, (NH)_2_), 3.34 (s, 4H, (CH_2_)_2_); ^13^C-NMR (δ, DMSO-*d*_6_): 129.3 (C-2, C-3, C-5, C-6), 136.7 (C-1, C-4); 161.0 (C-1′, C-3′), 105 (C-2′), 149.8 (C-5′) pyrimidine; 142.0 (C-1″), 126.4 (C-2″, C-6″), 124.5 (C-3″, C-5″), 147.4 (C-4″); 144.5 (C-1‴), 115.9 (C-3‴, C-5‴), 119.0 (C-2‴, C-6‴), 159.8 (C-5‴); 169.2 (C=O), 68.0 (CH_2_); Elem. Anal. Calcd. C_42_H_30_F_2_N_10_O_6_: C, 62.37; H, 3.74; N, 17.32; Found: C, 62.33; H, 3.72; N, 17.35; MS ES + (ToF): *m/z* 810 [M^+^ +1].

##### N,N′-(6,6′-(1,4-Phenylene)bis(4-(4-nitrophenyl)pyrimidine-6,2-diyl))bis(2-((2-fluorophenyl) amino)acetamide) (12)

Gold Yellow crystals; M.p.: 170–172 °C; R*f* value: 0.25; Yield: 77.12%; IR (KBr pellets, cm^−1^): 3108 (C–H str., phenyl nucleus), 1519 (C=C str., phenyl nucleus), 1665 (NH–C=O str.), 1600 (N=CH str., of pyrimidine), 2934 (C–H str., CH_2_), 1211 (C–NH– str.), 1340 (C–N sym. str., Ar–NO_2_), 1106 (C–F str., Ar–F); ^1^H-NMR (δ, DMSO-*d*_6_): 7.91–839 {m, 20H, Ar = H-2, H-3, H-5, H-6, (H-2″, H-3″, H-5″, H-6″) × 2, (H-3‴, H-4‴, H-5‴, H-6‴) × 2}, 7.90 (s, 2H, (CH)_2_ of pyrimidine), 3.38 (s, 4H, (CH_2_), 8.00 (s, 2H, (NH)_2_); ^13^C-NMR (δ, DMSO-*d*_6_): 129.3 (C-2, C-3, C-5, C-6), 135.5 (C-1, C-4); 162.0 (C-1′, C-3′), 105 (C-2′), 149.7 (C-5′) pyrimidine; 127.5 (C-2″, C-6″), 124.7 (C-3″, C-5″), 147.6 (C-4″); 130.5 (C-1‴), 153.2 (C-3‴), 116.9 (C-3‴), 121.9 (C-4‴); 188.2 (C=O), 49.0 (CH_2_); Elem. Anal. Calcd. C_42_H_30_F_2_N_10_O_6_: C, 62.37; H, 3.74; N, 17.32; Found; C, 62.34; H, 3.67; N, 17.28; MS ES + (ToF): *m/z* 810 [M^+^ +1].

##### N,N′-(6,6′-(1,4-Phenylene)bis(4-(4-nitrophenyl)pyrimidine-6,2-diyl))bis(2-((4-chloro-2-nitro phenyl)amino)acetamide) (13)

Light yellow crystals; M.p.: 175–177 °C; R*f* value: 0.16; Yield: 85.25%; IR (KBr pellets, cm^−1^): 3107 (C–H str., phenyl nucleus), 1519 (C=C str., phenyl nucleus), 1665 (NH–C=O str.), 1600 (N=CH str., of pyrimidine), 2936 (C–H str., CH_2_), 1247 (C–NH– str.), 1341 (C–N sym. str., Ar–NO_2_), 761 (C–Cl str., Ar–Cl); ^1^H-NMR (δ, DMSO-*d*_6_): 7.05–8.39 {m, 18H, Ar = H-2, H-3, H-5, H-6, (H-2″, H-3″, H-5″, H-6″) × 2, (H-3‴, H-5‴, H-6‴) × 2}, 7.48 (s, 2H, (CH)_2_ of pyrimidine), 8.00 (s, 2H, (NH)_2_), 3.66 (s, 4H, (CH_2_)_2_); ^13^C-NMR (δ, DMSO-*d*_6_): 128.9 (C-2, C-3, C-5, C-6), 135.5 (C-1,C-4); 165.0 (C-1′, C-3′), 110 (C-2′), 149.8 (C-5′) pyrimidine; 141.0 (C-1″), 126.4 (C-2″, C-6″), 124.0 (C-3″, C-5″), 149.9 (C-4″); 143.5 (C-1‴), 132.2 (C-2‴), 126.5 (C-3‴), 122.9 (C-4‴), 134.1 (C-5‴), 118.1 (C-6‴); 188.2 (C=O), 52.5 (CH_2_); Elem. Anal. Calcd. C_42_H_28_Cl_2_N_12_O_10_: C, 54.15; H, 3.03; N, 18.04; Found: C, 54.13; H, 3.00; N, 18.01; MS ES + (ToF): *m/z* 933 [M^+^ +1].

##### N,N′-(6,6′-(1,4-Phenylene)bis(4-(4-nitrophenyl)pyrimidine-6,2-diyl))bis(2-((2,6-dimethyl phenyl)amino)acetamide) (14)

Gold yellow crystals; M.p.: 158–160 °C; R*f* value: 0.38; Yield: 82.33%; IR (KBr pellets, cm^−1^): 3074 (C–H str., phenyl nucleus), 1520 (C=C str., phenyl nucleus), 1692 (NH–C=O str.), 1601 (N=CH str., of pyrimidine), 2853 (C–H str., CH_2_), 1211 (C–NH– str.), 1343 (C–N sym. str., Ar–NO_2_);^1^H-NMR (δ, DMSO-*d*_6_): 7.94–8.39 {m, 18H, Ar = H-2, H-3, H-5, H-6, (H-2″, H-3″, H-5″, H-6″) × 2, (H-3‴, H-4‴, H-5‴) × 2}, 7.91 (s, 2H, (CH)_2_ of pyrimidine), 8.01 (s, 2H, (NH)_2_), 3.39 (s, 4H, (CH_2_)_2_), 2.09 (s, 6H, (CH_3_)_2_); ^13^C-NMR (δ, DMSO-*d*_6_): 128.6 (C-2, C-3, C-5, C-6), 135.5 (C-1, C-4); 162.0 (C-1′, C-3′), 108 (C-2′), 149.8 (C-5′) pyrimidine; 141.0 (C-1″), 126.5 (C-2″, C-6″), 123.5 (C-3″, C-5″), 149.0 (C-4″); 145.5 (C-1‴), 119.0 (C-4‴), 127.2 (C-2‴, C-6‴), 127.9 (C-3‴, C-5‴); 170.2 (C=O), 52.5 (CH_2_), 17.8 (2,6-CH_3_); Elem. Anal. Calcd. C_46_H_40_N_10_O_6_: C, 66.66; H, 4.86; N, 16.90; Found: C, 66.63; H, 4.81; N, 16.87; MS ES + (ToF): *m/z* 830 [M^+^ +1].

##### N,N′-(6,6′-(1,4-Phenylene)bis(4-(4-nitrophenyl)pyrimidine-6,2-diyl))bis(2-((2,4-dimethyl phenyl)amino)acetamide) (15)

Gold yellow crystals; M.p.: 135–137 °C; R*f* value: 0.45; Yield: 89.63%; IR (KBr pellets, cm^−1^): 3074 (C–H str., phenyl nucleus), 1519 (C=C str., phenyl nucleus), 1664 (NH–C=O str.), 1597 (N=CH str., of pyrimidine), 2917 (C–H str., CH_2_), 1208 (C–NH– str.), 1339 (C–N sym. str., Ar–NO_2_); ^1^H-NMR (δ, DMSO-*d*_6_): 6.58-8.40 {m, 18H, Ar = H-2, H-3, H-5, H-6, (H-2″, H-3″, H-5″, H-6″) × 2, (H-3‴, H-5‴, H-6‴) × 2}, 7.47 (s, 2H, (CH)_2_ of pyrimidine), 8.00 (s, 2H, (NH)_2_), 3.36 (s, 4H, (CH_2_)_2_), 2.51(s, 6H, (CH_3_)_2_); ^13^C-NMR (δ, DMSO-*d*_6_): 128.9 (C-2, C-3, C-5, C-6), 136.5 (C-1, C-4); 163.0 (C-1′, C-3′), 104 (C-2′), 149.8 (C-5′) pyrimidine; 141.0 (C-1″), 126.4 (C-2″, C-6″), 124.5 (C-3″, C-5″), 149.8 (C-4″); 143.6 (C-1‴), 126.5, 126.2 (C-2‴, C-5‴), 133.9 (C-3‴), 136.9 (C-4‴), 116.1 (C-6‴); 188.2 (C=O), 52.0 (CH_2_), 18.2 (*o*-CH_3_), 21.2 (*p*-CH_3_); Elem. Anal. Calcd. C_46_H_40_N_10_O_6_: C, 66.66; H, 4.86; N, 16.90; Found: C, 66.63; H, 4.87; N, 16.95; MS ES + (ToF): *m/z* 830 [M^+^ +1].

##### N,N′-(6,6′-(1,4-Phenylene)bis(4-(4-nitrophenyl)pyrimidine-6,2-diyl))bis(2-((4-bromophenyl) amino)acetamide) (16)

Gold yellow crystals; M.p.: 138–140 °C; R*f* value: 0.23; Yield: 87.77%; IR (KBr pellets, cm^−1^): 3107 (C–H str., phenyl nucleus), 1519 (C=C str., phenyl nucleus), 1665 (NH–C=O str.), 1599 (N=CH str., of pyrimidine), 2933 (C–H str., CH_2_), 1209 (C–NH– str.), 1341 (C–N sym. str., Ar–NO_2_), 697 (C–Br str., Ar–Br); ^1^H-NMR (δ, DMSO-*d*_6_): 6.51–8.69 {m, 20H, Ar = H-2, H-3, H-5, H-6, (H-2″, H-3″, H-5″, H-6″) × 2, (H-2‴, H-3‴, H-5‴, H-6‴) × 2}, 7.55 (s, 2H, (CH)_2_ of pyrimidine), 8.00 (s, 2H, (NH)_2_), 3.37 (s, 4H, (CH_2_)_2_); ^13^C-NMR (δ, DMSO-*d*_6_): 128.2 (C-2, C-3, C-5, C-6), 136.7 (C-1, C-4); 160.0 (C-1′, C-3′), 104 (C-2′), 149.8 (C-5′) pyrimidine; 141.5 (C-1″), 126.4 (C-2″, C-6″), 123.5 (C-3″, C-5″), 149.7 (C-4″); 145.5 (C-1‴), 115 (C-2‴, C-6‴), 132.0 (C-3‴, C-5‴), 118.8 (C-5‴); 188.2 (C = O), 52.5 (CH_2_); Elem. Anal. Calcd. C_42_H_30_Br_2_N_10_O_6_: C, 54.21; H, 3.25; N, 15.05; Found: C, 54.18; H, 3.20; N, 15.00; MS ES + (ToF): *m/z* 933 [M^+^ +1].

##### N,N′-(6,6′-(1,4-Phenylene)bis(4-(4-nitrophenyl)pyrimidine-6,2-diyl))bis(2-((2,3-dichlorophenyl) amino)acetamide) (17)

Gold yellow crystals; M.p.: 195–197 °C; R*f* value: 0.16; Yield: 72.01%; IR (KBr pellets, cm^−1^): 3108 (C–H str., phenyl nucleus), 1520 (C=C str., phenyl nucleus), 1666 (NH–C=O str.), 1599 (N=CH str., of pyrimidine), 2934 (C–H str., CH_2_), 1211 (C–NH– str.), 1341 (C–N sym. str., Ar–NO_2_), 700 (C–Cl str., Ar–Cl); ^1^H-NMR (δ, DMSO-*d*_6_): 7.93–8.39 {m, 18H, Ar = H-2, H-3, H-5, H-6, (H-2″, H-3″, H-5″, H-6″) × 2, (H-4‴, H-5‴, H-6‴) × 2}, 7.91 (s, 2H, (CH)_2_ of pyrimidine), 8.00 (s, 2H, (NH)_2_), 3.38 (s, 4H, (CH_2_)_2_); ^13^C-NMR (δ, DMSO-*d*_6_): 128.9 (C-2, C-3, C-5, C-6), 135.1 (C-1, C-4); 162.0 (C-1′, C-3′), 104.1 (C-2′), 149.0 (C-5′) pyrimidine; 126.4 (C-2″, C-6″), 123.8 (C-3″, C-5″), 149.6 (C-4″); 145.3 (C-1‴), 123.6 (C-2‴), 133.9 (C-3‴), 122.9 (C-4‴), 129.1 (C-5‴), 113.1 (C-6‴); 188.2 (C=O), 52.6 (CH_2_); Elem. Anal. Calcd. C_42_H_28_Cl_4_N_10_O_6_: C, 55.40; H, 3.10; N, 15.38; Found: C, 55.38; H, 3.07; N, 15.33; MS ES + (ToF): *m/z* 912 [M^+^ +1].

##### N,N′-(6,6′-(1,4-Phenylene)bis(4-(4-nitrophenyl)pyrimidine-6,2-diyl))bis(2-((2,6-dichlorophenyl) amino)acetamide) (18)

Gold yellow crystals; M.p.: 129–131 °C; R*f* value: 0.33; Yield: 75.30%; IR (KBr pellets, cm^−1^): 3107 (C–H str., phenyl nucleus), 1520 (C=C str., phenyl nucleus), 1666 (NH–C = O str.), 1599 (N=CH str., of pyrimidine), 2936 (C–H str., CH_2_), 1211 (C–NH– str.,), 1342 (C–N sym. str., Ar–NO_2_), 700 (C–Cl str., Ar–Cl); ^1^H-NMR (δ, DMSO-*d*_6_): 7.04–9.09 {m, 18H, Ar = H-2, H-3, H-5, H-6, (H-2″, H-3″, H-5″, H-6″) × 2, (H-3‴, H-4‴, H-5‴) × 2}, 7.75 (s, 2H, (CH)_2_ of pyrimidine), 8.01 (s, 2H, (NH)_2_), 3.38 (s, 4H, (CH_2_)_2_); ^13^C-NMR (δ, DMSO-*d*_6_): 127.3 (C-2, C-3, C-5, C-6), 134.5 (C-1, C-4); 164.0 (C-1′, C-3′), 102 (C-2′), 148.3 (C-5′) pyrimidine; 126.4 (C-2″, C-6″), 124.5 (C-3″, C-5″), 148.6 (C-4″); 144.5 (C-1‴), 123.5 (C-2‴, C-6‴), 128.5 (C-3‴, C-5‴), 121.1 (C-6‴); 165.2 (C=O), 53.0 (CH_2_); Elem. Anal. Calcd. C_42_H_28_Cl_4_N_10_O_6_: C, 55.40; H, 3.10; N, 15.38; Found: C, 76.13; H, 4.24; N, 11.12; MS ES + (ToF): *m/z* 912 [M^+^ + 1].

##### N,N′-(6,6′-(1,4-Phenylene)bis(4-(4-nitrophenyl)pyrimidine-6,2-diyl))bis(2-((3-methoxyphenyl) amino)acetamide) (19)

Gold yellow crystals; M.p.: 198–200 °C; R*f* value: 0.21; Yield: 75.30%; IR (KBr pellets, cm^−1^): 3074 (C–H str., phenyl nucleus), 1519 (C=C str., phenyl nucleus), 1666 (NH–C=O str.), 1598 (N=CH str., of pyrimidine), 2836 (C–H str., CH_2_), 1210 (C–NH– str.), 1341 (C–N sym. str., Ar–NO_2_), 1105 (C–O–C str., Ar–OCH_3_); ^1^H-NMR (δ, DMSO-*d*_6_): 5.22–8.69 {m, 20H, Ar = H-2, H-3, H-5, H-6, (H-2″, H-3″, H-5″, H-6″) × 2, (H-2‴, H-4‴, H-5‴, H-6‴) × 2}, 7.41 (s, 2H, (CH)_2_ of pyrimidine), 8.01 (s, 2H, (NH)_2_), 3.73 (s, 4H, (CH_2_)_2_, 3.38 (s, 6H, (OCH_3_)_2_); ^13^C-NMR (δ, DMSO-*d*_6_): 128.3 (C-2, C-3, C-5, C-6), 136.7 (C-1, C-4); 160.0 (C-1′, C-3′), 106 (C-2′), 149.8 (C-5′) pyrimidine; 141.5 (C-1″), 126.4 (C-2″, C-6″), 123.5 (C-3″, C-5″), 148.3 (C-4″); 149.7 (C-1‴), 106.4 (C-2‴, C-6‴), 113.3 (C-5‴); 188.2 (C=O), 54.4 (CH_2_), 55.1 (OCH_3_); Elem. Anal. Calcd. C_44_H_36_N_10_O_8_: C, 63.46; H, 4.36; N, 16.82; Found: C, 63.39; H, 4.30; N, 16.86; MS ES + (ToF): *m/z* 834 [M^+^ +1].

##### N,N′-(6,6′-(1,4-Phenylene)bis(4-(4-nitrophenyl)pyrimidine-6,2-diyl))bis(2-(2-methoxyphenyl) acetamide (20)

Canary yellow crystals; M.p.: 179–180 °C; R*f* value: 0.32; Yield: 89.23%; IR (KBr pellets, cm^−1^): 3076 (C–H str., phenyl group), 1519 (C=C str., phenyl group), 1666 (NH–C=O str.), 1599 (N=CH str., of pyrimidine), 2842 (C–H str., CH_2_), 1211 (C–NH– str.), 1340 (C–N sym. str., Ar–NO_2_), 1107 (C–O–C str., Ar–OCH_3_); ^1^H-NMR (δ, DMSO-*d*_6_): 7.14-8.39 {m, 20H, Ar = H-2, H-3, H-5, H-6, (H-2″, H-3″, H-5″, H-6″) × 2, (H-3‴, H-4‴, H-5‴, H-6‴) × 2}, 7.47 (s, 2H, (CH)_2_ of pyrimidine), 8.00 (s, 2H, (NH)_2_), 3.82 (s, 4H, (CH_2_)_2_), 3.75 (s, 6H, (OCH_3_)_2_); ^13^C-NMR (δ, DMSO-*d*_6_): 128.9 (C-2, C-3, C-5, C-6), 136.7 (C-1, C-4); 164.0 (C-1′, C-3′), 110.0 (C-2′), 149.8 (C-5′) pyrimidine; 141.5 (C-1″), 126.4 (C-2″, C-6″), 124.5 (C-3″, C-5″), 149.7 (C-4″); 126.5 (C-1‴), 158.0 (C-2‴), 55.5 (OCH_3_), 113.3 (C-3‴), 128.9 (C-4‴), 121.3 (C-5‴), 133.2 (C-6‴); 188.2 (C=O), 39.0 (CH_2_); Elem. Anal. Calcd. C_44_H_36_N_10_O_8_: C, 63.46; H, 4.36; N, 16.82; Found: C, 63.40; H, 4.31; N, 16.85; MS ES + (ToF): *m/z* 834 [M^+^ +1].

#### Biological study (antimicrobial and anticancer)

The antimicrobial activity i.e. The minimum inhibitory concentration (MIC) of the synthesized compounds (**1**–**20**) was determined by tube dilution method [26] using cefadroxil (antibacterial) and fluconazole (antifungal) as reference drugs against Gram-positive [*S. aureus,* MTCC-3160 (Microbial Type Culture Collection); *B. subtilis,* MTCC-441] and Gram-negative bacteria (*E. coli,* MTCC-443; *P. aeruginosa,* MTCC-3542; *S. enterica,* MTCC-1165). The antifungal activity was assayed against yeast (*C. albicans,* MTCC-227) and mould (*A. niger*, MTCC-281). Serial dilutions of the test compounds and reference drugs were prepared in double strength nutrient broth I.P. (bacteria) or sabouraud dextrose broth I.P. (fungi) [27]. The stock solution of the test compounds and reference drugs was prepared in dimethyl sulfoxide (DMSO). Further progressive dilutions were done to obtain final concentrations of 50, 25, 12.5, 6.25, 3.125 and 1.562 µg/mL. The samples were incubated at 37 ± 1 °C for 24 h (bacteria), at 25 ± 1 °C for 7 days (*A. niger*) and at 37 ± 1 °C for 48 h (*C. albicans*) respectively and the results were recorded in terms of MIC. The MIC was the lowest concentration of the tested compound that yields no visible growth of microorganisms in the tube. To ensure that the solvent had no effect on the bacterial growth, a control was performed with the test medium supplemented with DMSO at the same dilutions as used in the experiments and DMSO had no effect on the microorganisms in the concentrations studied. The anticancer screening (IC_50_ = µmol/mL) of synthesized compounds was determined against human colorectal carcinoma [HCT-116 (ATCC (American Type Culture Collection) CCL-247)] cancer cell line using sulforhodamine-B (SRB) assay. In this study, the culture material was fixed with trichloroacetic acid and then stained for 30 min with 0.4% (w/v) sulforhodamine B mixed with 1% acetic acid. Unbound dye was discarded by five washes of 1% acetic acid solution and protein-bound dye was extracted with 10 m*M* Tris base [tris(hydroxymethyl) aminomethane] for confirmation of optical density in a computer-interfaced, 96-well microtiter plate reader [28].

## Conclusion

In summary, a series of new bis-pyrimidine acetamide molecules was synthesized in good yields and its chemical structures were confirmed by ^1^H/^13^C-NMR, Mass, FT-IR studies and elemental analyses. All the synthesized compounds were tested for their in vitro antimicrobial and anticancer potentials. Among the synthesized compounds, compounds, **3**, **13**, **16**, **17** and **18** exhibited good antimicrobial potential against different microorganism (bacterial species: *S. aureus*, *B. subtilis, E. coli, P. aeruginosa*, *S. enterica* and fungal species: *A. niger* and *C. albicans*) than the standard drugs cefadroxil and fluconazole. Similarly, compounds, **12**, **16** and **18** were found to be more effective against HCT 116 cancer cell line than the standard drug, 5-fluorouracil.
